# Effects of Photodegradation on the Optical Indices of Chromophoric Dissolved Organic Matter from Typical Sources

**DOI:** 10.3390/ijerph192114268

**Published:** 2022-11-01

**Authors:** Wan-E Zhuang, Wei Chen, Liyang Yang

**Affiliations:** 1 College of Life Sciences, Fujian Agriculture and Forestry University, Fuzhou 350002, China; 2 College of Environment and Safety Engineering, Fuzhou University, Fuzhou 350108, China

**Keywords:** CDOM, photodegradation, fluorescence EEMs-PARAFAC, absorption spectroscopy

## Abstract

Chromophoric dissolved organic matter (CDOM) plays important roles in aquatic environments, and its optical properties provide a series of indices for evaluating the source and composition of dissolved organic matter (DOM). However, little is known about the varying photodegradation of CDOM from different sources and the effects on the optical indices of DOM composition. This was studied for typical natural and anthropogenic sources (plant and leaf litter leachates, the influent and effluent of a wastewater treatment plant, and a river). The CDOM absorption (*a*_280_) showed a lower degradability for the plant leachate than other sources, mainly due to its low molecular weight and aromaticity. Four fluorescent components were identified with excitation–emission matrices-parallel factor analysis (EEMs-PARAFAC), namely benzoic acid/monolignol-like C1, humic-like C2 and C3, and tryptophan-like C4. The plant leachate contained mainly C1, which was photodegraded moderately, while other sources had more C2 and C3 with higher photodegradability. C4 was photodegraded in most sources but was photoproduced in the leaf litter leachate. The absorption slope (*S*_275–295_) and slope ratio (*S*_R_) increased while the humification index (HIX) decreased, suggesting a decreasing molecular weight and humic content by photodegradation. This was consistent with the decreasing %C2 and %C3 but increasing %C4, which indicated preferential removal of humic-like components. The %C1, %C2, biological index (BIX), and fluorescence index (FI) were less affected by photodegradation than other indices for most sources. These results have implications for a better understanding of the photochemistry of CDOM and the applications of optical indices.

## 1. Introduction

CDOM, the light absorbing fraction of DOM, is important in many processes of aquatic environments [1,2]. The absorbance of UV lights by CDOM protects the organisms from harmful radiation [3], while the absorbance of photosynthetic active radiation competes with the light availability for phytoplankton productivity [4]. CDOM is important in the cycling of elements such as carbon and nutrients [5,6,7]. It can also bind with pollutants and affect their speciation, behavior, and bioavailability [8,9,10]. The reaction of CDOM with disinfectants can produce harmful byproducts in drinking water treatments [11]. Therefore, it is important to study the biogeochemistry and underlying mechanisms of CDOM in aquatic environments.

The properties of CDOM in aquatic environments are dependent on a series of factors including the inputs from different sources and the biogeochemical transformations in the environments [1,2]. In particular, photodegradation is a critical process in processing CDOM which can produce inorganic carbon (CO_2_ and CO), low molecular weight compounds, and nutrients [5,12]. The photodegradation of CDOM is affected by both the initial CDOM property (e.g., aromaticity) and environmental factors [13,14,15,16,17]. In particular, the CDOM in aquatic environments originates from allochthonous and autochthonous sources with different compositions that result in different reactivity of CDOM [13,18,19]. Further, the allochthonous sources include not only natural sources but also anthropogenic sources (e.g., raw and treated wastewater) with distinct CDOM compositions [20]. It is warranted to study the different photodegradation behavior of CDOM from these typical sources for a better understanding of CDOM biogeochemistry.

The properties of CDOM are commonly characterized by absorption and fluorescence spectroscopy [1,2,21,22,23,24]. The absorption coefficient is indicative of the CDOM level, and the absorption slope and slope ratio are popular for inferring the changes in average molecular weight and aromaticity of DOM [25]. The fluorescence spectroscopy provides information for different components of fluorescent DOM (FDOM) and the indices for assessing the source and humic content of DOM [21,26]. In particular, the application of parallel factor analysis to fluorescence excitation–emission matrices (EEMs-PARAFAC) can identify different FDOM components accurately [22,23,24]. These optical indices from the absorption and fluorescence measurements are widely used for tracking the source, composition, and reactivity of CDOM in aquatic environments. The successful application of optical indices in the source tracking is complicated by the changes in indices due to photodegradation, although some indices may be more stable than others [27,28,29]. Thus, it is worth further testing the effects of photodegradation on the optical indices of CDOM from typical natural and anthropogenic sources for the appropriate applications of optical indices.

Therefore, the aim of this study was to: (1) examine the photodegradation behaviors of CDOM and different FDOM components from typical sources using the absorption spectroscopy and fluorescence EEMs-PARAFAC and (2) test the effects of photodegradation on the optical indices of DOM composition. The results would provide new insights into the variability of different CDOM components, diverse sources, and related optical indices under photodegradation conditions. These would be helpful for a better understanding of CDOM biogeochemistry and the applications of optical indices in the field of CDOM study.

## 2. Materials and Methods

### 2.1. Sample Collection and Photodegradation Experiment

The CDOM samples from typical sources of the subtropical Minjiang Watershed (SE China) in this study were similar to those in our previous study on their microbial degradation [20]. Briefly, two wastewater samples were collected from the influent and effluent of a local municipal wastewater treatment plant in Fuzhou City. The plant and leaf litter leachates were prepared by leaching the typical herbaceous plants (Pteridophyta, at a solid-to-solution ratio of 1:10) and the corresponding leaf litter (solid-to-solution ratio: 1:5) with Milli-Q water at 25 °C for 24 h. The river sample was collected from the mainstem of Minjiang River (SE China, at 26°6′24.23″ N, 119°11′27.48″ E). All CDOM samples were filtered sequentially through pre-combusted Whatman 0.7 μm GF/F and acid-rinsed 0.2 μm Millipore polycarbonate filters for the photodegradation experiment.

Photodegradation of CDOM was performed for 6 days, following the procedure described previously [25,30]. The local daily solar radiation was 6.8–24.3 MJ m^−2^ (mean: 18.2 ± 6.3 MJ m^−2^) during this period (https://www.xihe-energy.com (accessed on 7 September 2022)). The CDOM filtrates were transferred into several pre-combusted quartz tubes (with a volume of 125 mL for each quartz tube), placed in a tap water tank, and irradiated under natural sunlight on the roof of our college building at Fuzhou University. Half of the tubes were wrapped in aluminum foil as the dark group, which showed limited changes and are not discussed below. Samples were taken for measurements at the beginning and after 1, 3, and 6 days (i.e., after 10, 30, and 60 h solar radiation) for optical measurements. All measurements were performed in triplicate, and the mean analytical precision was 3.2% for the optical indices in this study.

### 2.2. Sample Measurements and Analysis

The CDOM absorption spectra were scanned over 240–800 nm with a Shimadzu UV-1780 UV–Vis spectrometer and were corrected for the baseline fluctuation by subtracting the mean absorbance over 700–800 nm [20]. The absorption coefficient at 280 nm (*a*_280_) was used as an index for the CDOM level [1,2,7]. *a*_280_ correlated strongly with the absorption coefficient at 254 nm, another index for aromatic organic matter that was sensitive to photodegradation (*a*_254_ = 1.37 × *a*_280_ + 1.40, *r* = 0.952). The absorption slope over 275–295 nm (*S*_275–295_) and slope ratio (*S*_R_), which correlated negatively with the aromaticity and average molecular weight of organic matter, were also calculated following the procedure reported previously [25].

Fluorescence EEMs were scanned for the CDOM samples over excitation (Ex) wavelengths of 240–450 nm and emission (Em) wavelengths of 280–600 nm using a Cary Eclipse fluorescence spectrophotometer following the procedure described previously [20,30]. The inner-filter effect was corrected for each EEM with the absorption spectra for the same sample [31], and the blank signals of Milli-Q water were subtracted. The corrected EEMs were modeled with PARAFAC using the DOMFluor toolbox 1.7 [20,32]. The number of PARAFAC components was determined with split-half validation. The fluorescence intensity (*F*_max_) of each component was used to represent its level (in Raman units, RU), and the contribution of each component to the total fluorescence (%C1–%C4) was used as an index for the FDOM composition. Three popular indices were also calculated from the EEMs, namely the humification index (HIX), biological index (BIX), and fluorescence index (FI). HIX is the ratio of fluorescence over Em 434–480 nm to that over 300–346 nm (at Ex 255 nm), which is an index for the humic content or humification degree of DOM [33]. BIX is the ratio of fluorescence at Em 380 nm to that at 430 nm (at Ex 310 nm), which is an index for the source of DOM [26]. FI is the ratio of fluorescence at Em 470 nm to that at 520 nm (at Ex 370 nm), which is another index for the DOM source [34].

The decreases of *a*_280_ and the *F*_max_ values of PARAFAC components during photodegradation were fitted with the exponential decay model [16,35]:

*Y*_t_ = *Y*_1_ × *e*^−*kt*^ + *Y*_2_ (1)

where *Y*_t_ is the modeled level at time *t*, and *Y*_1_, *k*, and *Y*_2_ are adjustable parameters that represent the photodegradable component at the beginning, the decay rate, and the nonphotoreactive component, respectively. Correspondingly, the photodegradable fraction (*f*) was calculated as:

*f* = *Y*_1_/(*Y*_1_ + *Y*_2_) × 100% (2)

The half-life (*t*_1/2_) of the photodegradable component was calculated as:

*t*_1/2_ = ln(2)/*k* (3)

## 3. Results

### 3.1. Absorption Results

**Figure 1:**
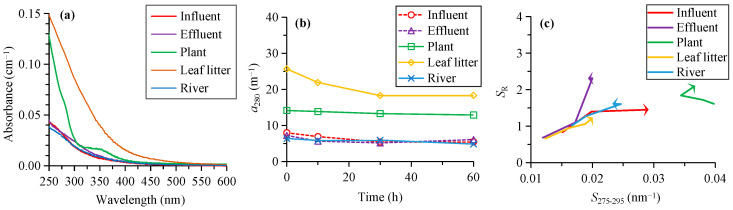
(**a**) The absorbance spectra of CDOM from different sources, (**b**) changes in the absorption coefficient of CDOM (*a*_280_) with the irradiation time, and (**c**) changes in the spectral slope (*S*_275–295_) and slope ratio (*S*_R_) by the irradiation, with the arrows showing the direction of changes.

The absorbance of CDOM generally decreased with the increase in wavelength for all sources, except for the plant leachate that showed a shoulder peak at around 350 nm (Figure 1a). The initial *a*_280_ ranged from 6.4 for the river water to 25.6 m^−1^ for the leaf litter leachate (Figure 1b). It decreased by 9%–31% to 4.8–18.3 m^−1^ after the 60 h photodegradation (Figure 1b; Table 1).

The initial *S*_275–295_ ranged from 0.0119 to 0.0123 nm^−1^ for the effluent and leaf litter leachate, to 0.0151–0.0167 nm^−1^ for the influent and river, and 0.0398 nm^−1^ for the plant leachate (Figure 1c). The *S*_275–295_ increased by 40%–92% for most sources after photodegradation, while it decreased by 8% for the plant leachate (Table 1). The final *S*_275–295_ ranged from 0.0193 to 0.0200 nm^−1^ for the effluent and leaf litter leachate, to 0.0233 to 0.0291 nm^−1^ for the influent and river and 0.0367 nm^−1^ for the plant leachate (Figure 1c).

The initial *S*_R_ was lowest (0.66–0.68) for the effluent and leaf litter leachate, which increased to 0.83–1.02 for the influent and river and 1.61 for the plant leachate (Figure 1c). The *S*_R_ increased by 31%–237% for all sources after photodegradation (Table 1). The final *S*_R_ ranged from 1.23 for the leaf litter leachate, to 1.45 to 1.62 for the influent and river, and 2.10–2.30 for the effluent and plant leachate (Figure 1c).

### 3.2. Fluorescence Results

**Figure 2:**
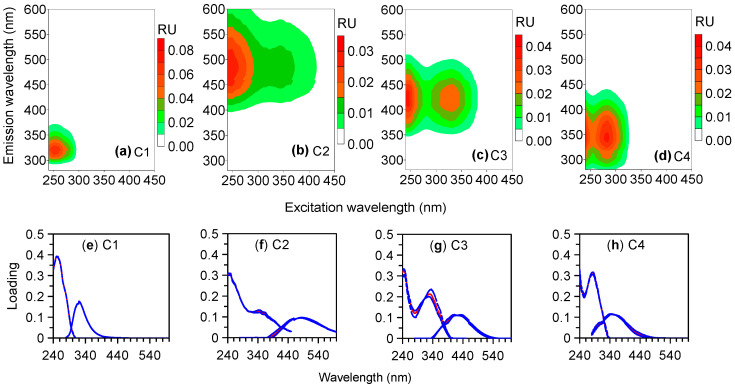
(**a**–**d**) EEM contours of the four fluorescent components (C1–C4) identified with PARAFAC in this study and (**e**–**h**) their excitation and emission loadings based on the whole dataset (red solid lines) and random halves in split-half analysis (blue dotted lines).

Four components were identified from the fluorescence EEMs using PARAFAC in this study (C1–C4, Figure 2), which were compared with those in the OpenFluor database [36]. C1 showed one Ex maximum at 250 nm and one Em maximum at 320 nm, which was similar to the fluorescence of benzoic acid [37] and monolignol [38]. C2 displayed Ex/Em maxima at 250/485 nm, resembling a terrestrial humic-like component previously identified (C1 in [39,40]). C3 had two Ex maxima at 245 and 330 nm and one Em maximum at 412 nm, similar to the microbial humic-like component (C1 in [41]; C2 in [42]). C4 had Ex/Em maxima at (240, 285)/342 nm, which resembled the tryptophan-like component (C5 in [43]; C4 in [44]).

**Figure 3:**
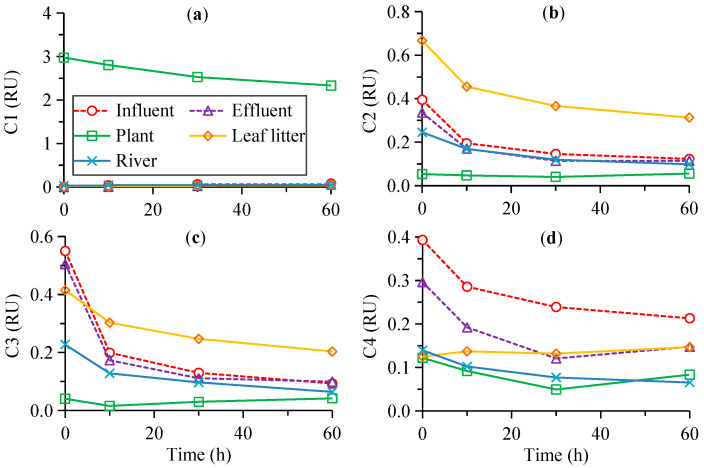
Changes in the fluorescence intensity of PARAFAC components (C1–C4, **a**–**d**) with the irradiation time.

The initial *F*_max_ of C1 was up to 2.97 RU for the plant leachate, while it was as low as 0–0.03 RU for other sources (Figure 3a). C1 in the plant leachate decreased by 22% to 2.33 RU after photodegradation (Figure 3a; Table 1). The initial *F*_max_ values of C2 and C3 were 0.25–0.67 and 0.23–0.55 RU for most sources, except for the lowest values of 0.04–0.05 RU for the plant leachate that is not discussed below (Figure 3b,c). C2 and C3 decreased by 53%–69% and 51%–83% to 0.10–0.31 and 0.06–0.20 RU after photodegradation, respectively (Figure 3b,c; Table 1). The initial *F*_max_ of C4 was higher for the influent and effluent (0.30–0.39 RU) than for other sources (0.12–0.14 RU, Figure 3d). It decreased by 32%–53% for most sources but increased by 17% for the leaf litter leachate after photodegradation (Table 1). The final *F*_max_ of C4 ranged from 0.07 to 0.08 RU for the plant leachate and river, and 0.21 RU for the influent (Figure 3d).

**Figure 4:**
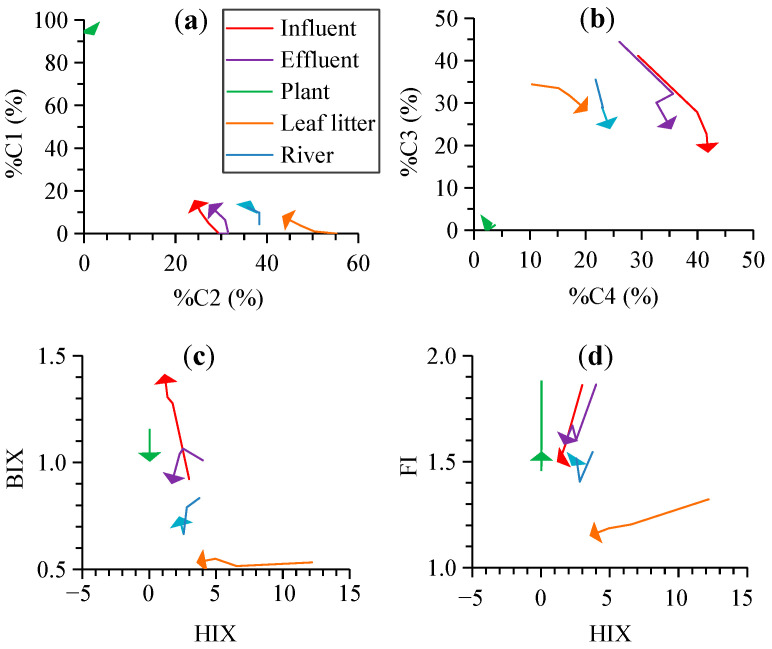
Changes in the fluorescence indices for the chemical composition of DOM by photodegradation ((**a**,**b**): %C1–%C4; (**c**,**d**): HIX, BIX, and FI), with the triangles at the end of the lines showing the direction of changes.

The initial %C1 was highest for the plant leachate (93.2%), which showed minor changes to 92.8% after the 60 h photodegradation (Figure 4a; Table 1). The initial %C1 was low for other sources (0%–4.4%) and increased to final values of 8.2%–15.7%. The values of %C2–%C4 were low for the initial plant leachate (1.3%–3.8%) and were generally stable (1.7%–3.3%) after photodegradation (Figure 4a,b). The %C2 for other sources decreased from 29.5%–55.2% to 24.1%–43.3%, and %C3 decreased from 34.4%–44.4% to 18.3%–28.2% after photodegradation. The %C4 for other sources increased from 10.4%–29.4% to 20.3%–41.9% after photodegradation.

The initial HIX was lowest for the plant leachate (0.04), which was stable in the photodegradation experiment (0.03–0.04, Figure 4c). It was highest for the initial leaf litter leachate (12.2) that decreased by 71% to 3.5 after photodegradation. The initial HIX was 3.0–4.0 for other sources, which decreased by 40%–61% to 1.2–2.2. The initial BIX was much lower for the leaf litter leachate (0.53) than for other sources (0.83–1.16, Figure 4c). It increased by 53% to 1.41 for the influent, remained stable for the leaf litter leachate, and decreased by 10%–13% for other sources after photodegradation (Figure 4c, Table 1). The initial FI ranged from 1.32 for the leaf litter leachate to 1.55 to 1.56 for the plant leachate and river and 1.86 for the influent and effluent (Figure 4d). It decreased by 1%–19% to 1.15 for the leaf litter leachate and 1.48–1.58 for other sources after photodegradation (Figure 4d, Table 1).

### 3.3. The Degradation Model Results

**Figure 5:**
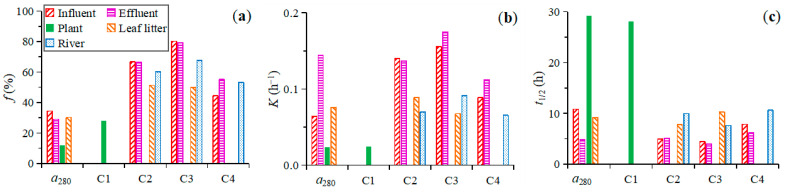
The photodegradable fraction (*f*, (**a**)), decay rate (*k*, (**b**)), and half-life (*t*_1/2_, (**c**)) of the bulk CDOM (*a*_280_) and each fluorescent component (C1–C4) from different sources.

The photodegradable fraction (*f*) of *a*_280_ ranged from 12.0% for the plant leachate to 29.0% to 34.4% for other sources (except for the river *a*_280_, which did not fit well with the model, Figure 5a). The degradation rate constant (*k*) of *a*_280_ was lowest for the plant leachate (0.024 h^−1^), which increased to 0.064–0.076 h^−1^ for the influent and leaf litter leachate and 0.144 h^−1^ for the effluent (Figure 5b). Correspondingly, the half-life (*t*_1/2_) of *a*_280_ was longest for the plant leachate (29.2 h), followed by those for the leaf litter leachate and influent (9.2–10.8 h), and was shortest for the effluent (4.8 h, Figure 5c).

The *f* of C1 was 28.2% for the plant leachate, with a low *k* of 0.025 h^−1^ and a high *t*_1/2_ of 28.1 h (Figure 5). The *f* of C2 and C3 were lowest for the leaf litter leachate (51.3% and 49.8%), which increased to 60.1%–67.6% for the river and were highest for the influent and effluent (66.5%–66.7% and 79.4%–80.3%). The *k* of C2 and C3 were lower for the leaf litter leachate and river (0.068–0.091 h^−1^) than for the influent and effluent (0.137–0.175 h^−1^). Correspondingly, the *t*_1/2_ of C2 and C3 were higher for the leaf litter leachate and river (7.6–10.2 h) than for the influent and effluent (4.0–5.1 h). The *f* of C4 was lower for the influent (44.4%) than for the effluent and river (53.3%–55.1%), while C4 did not fit the exponential decay modeling for the plant and leaf litter leachates. The *k* of C4 was higher for the effluent (0.112 h^−1^) than for the river and influent (0.065–0.089 h^−1^). Correspondingly, the *t*_1/2_ of C4 was shorter for the effluent (6.2 h) than for the influent (7.8 h) and river (10.6 h).

## 4. Discussion

### 4.1. Variability of Photodegradation of CDOM and Different Fluorescent Components from Typical Sources

Photodegradation is one of the most important processes in the biogeochemical cycles of organic matter. The results of this study revealed the variability of photodegradation of CDOM and FDOM from typical natural and anthropogenic sources. The CDOM level showed a weaker decrease for the plant leachate after the 60 h photodegradation (9% of initial *a*_280_) than for other sources including the influent, effluent, leaf litter leachate, and river (16%–31%, Table 1). Correspondingly, the photodegradable fraction of *a*_280_ was lower for the plant leachate with a lower degradation rate constant and longer half-life (Figure 5). This might be related to the different composition of CDOM between the plant leachate and other sources, since the samples from all sources were subject to similar solar irradiation in the same period. Generally, the initial plant leachate had higher *S*_275–295_ (0.0398 nm^−1^) and *S*_R_ (1.61) but lower HIX (0.04) than other sources (0.0119–0.0167 nm^−1^, 0.66–1.02, and 3.0–12.2, respectively, Figure 1 and Figure 4), which indicated lower molecular weight and humic content for the fresh plant leachate [25,26]. This is consistent with previous studies showing more effective photodegradation for the aromatic, unsaturated, and high molecular weight CDOM [15,27,45,46]. The difference in the initial CDOM level might be another factor [7,13]. The initial CDOM absorption of the plant leachate (14.1 m^−1^) was within the range of other sources (6.4–25.6 m^−1^, Table 1), indicating moderate light absorption for the plant leachate. Thus, the effect of the initial CDOM level probably only played a secondary role in the weaker photodegradation of CDOM in the plant leachate.

The difference in the photodegradability of different sources was also evident for the PARAFAC components. C1, which was the dominant component (up to 2.97 RU) in the plant leachate but was almost absent in other sources (0–0.03 RU), decreased by 22% in the plant leachate after photodegradation (Figure 3, Table 1). In contrast, the plant leachate contained little humic-like C2 and C3 (0.04–0.05 RU), which were more abundant (0.23–0.67 RU) and decreased by up to 51%–83% for other sources after photodegradation. Similarly, the strong photodegradation of humic-like components is reported for aquatic environments in previous studies [16,17,19,46]. The corresponding photodegradable fraction was lower for C1 of the plant leachate with a lower degradation rate constant and longer half-life than for C2 and C3 of other sources (Figure 5). Our results clearly showed that the FDOM from different sources contained different components with different photodegradability. Further, although the tryptophan-like C4 was present in all sources, it decreased (32%–53%) for most sources but increased (17%) for the leaf litter leachate after photodegradation. The removal of the tryptophan-like component by photodegradation is commonly reported previously, although it can also be released from the degradation of high molecular weight constituents [17,18,30]. In addition, the photodegradability of C2–C4 was generally higher for the anthropogenic sources (influent and effluent) than for the natural sources (the plant and leaf litter leachates, Table 1). These results indicated the different photochemical behavior of a similar component for different sources and the FDOM from the wastewater could be removed effectively by photodegradation. Such changes in the photodegradability of a similar component might be related to the initial CDOM property (e.g., different functional groups at similar wavelengths and different levels of FDOM and their precursors) and/or different environmental factors (e.g., inorganic ions such as iron) [7,9,13,14,15,16,17,18].

### 4.2. Effects of Photodegradation on the Optical Indices for the DOM Composition

The photodegradation of CDOM and FDOM as discussed above was accompanied by notable changes in most optical indices for the chemical composition of DOM (Figure 1 and Figure 4; Table 1). The *S*_275–295_ and *S*_R_ increased substantially (31%–237%) for most sources after photodegradation, except for the slight decrease in *S*_275–295_ for the plant leachate (8%, Table 1). These results indicated a general decrease in the average molecular weight of DOM by photodegradation, which is consistent with the findings in previous studies [25,27,30]. The slight decrease in *S*_275–295_ for the plant leachate was not consistent with the increasing *S*_R_, which might be related to the weak photodegradation of CDOM (9%) that resulted in less evident changes in the molecular weight. %C1 was stable in the photodegradation for the plant leachate (Table 1). %C2 and %C3 both decreased by photodegradation for all sources, although %C2 was less affected than %C3 (by 5%–22% vs. 18%–56%, Table 1). In contrast, %C4 increased for most sources including the influent, effluent, leaf litter leachate, and river (by 12%–96%), except for a slight decrease for the plant leachate (14%). These results indicated preferential removal of humic-like components (C2 and C3) over the protein-like C4 [16,17]. Correspondingly, the HIX decreased by up to 40%–71% for all sources except for the plant leachate, which was indicative of decreasing humic content of DOM [26,27]. In contrast, the BIX and FI were more stable in the photodegradation for most sources, which decreased by 0–15%. One exception was the influent that showed a notable increase in BIX (53%) and a moderate decrease in FI (19%).

Overall, our results demonstrated the notable effects of natural solar radiation on the DOM composition. On one hand, the results above demonstrated that absorption and fluorescence spectroscopy provided valuable indices for effectively detecting the effects of photodegradation on DOM. On the other hand, such changes in optical indices should be considered in their applications for tracking the sources of DOM under solar radiation conditions in natural environments. For example, the changes in end member values by photodegradation would affect the quantitative assessment of the contribution from different DOM sources, although the qualitative discrimination could be achieved based on different values of the optical indices for different sources (Figure 1c and Figure 4) [28,29]. In particular, the *S*_275–295_, *S*_R_, %C3, %C4, and HIX were more sensitive to photodegradation (Figure 1 and Figure 4, Table 1). The %C1, %C2, BIX, and FI were generally less affected, although the large increase in the influent BIX (53%) and the narrow range of FI for all sources (1.15–1.58) after photodegradation might partly limit their applications.

It is noteworthy that our photodegradation experiment was performed in the upper layer of the clear tap water bath. Thus, the results were representative of photodegradation in the surface water of clear waterbodies, providing information for comparing the photodegradability of different sources and different components and for evaluating the changes of optical indices for the DOM composition. In the field environments, the decreasing light availability with the increasing water depth or turbidity can weaken the photodegradation, although this could be compensated if the water residence time is longer such as in reservoirs, lakes, or long rivers. In addition, most microbes were excluded by filtration in the photodegradation experiment of this study. The interactions with microbial processes in the field may also amplify the implications of CDOM photodegradation. For example, it is reported that the photodegradation of refractory humic-rich DOM generally increased the bioavailability of DOM while the photodegradation of biologically reactive DOM may have a contrasting effect [18,47,48]. Therefore, the lower molecular weight compounds produced from photodegradation, as indicated by the increasing *S*_275–295_ and *S*_R_ for both natural (leaf litter) and anthropogenic (wastewater) sources in this study, might provide the substrates for microbial utilization, which would result in a coupling of photochemical and microbial degradation.

## 5. Conclusions

The photodegradation of CDOM and FDOM were different for typical sources. The CDOM of the plant leachate had a lower degradability than other natural and anthropogenic sources, mainly related to its low molecular weight and aromaticity. The dominant component C1 in the plant leachate showed moderate photodegradation, while the humic-like C2 and C3 were more abundant and more photodegradable in other sources. The tryptophan-like C4 was photodegraded in most sources but was photoproduced in the leaf litter leachate.

The optical indices for the DOM composition were also modified by photodegradation. The increasing *S*_275–295_ and *S*_R_ but decreasing HIX indicated a decreasing molecular weight and humic content of DOM for most sources by photodegradation. The decreasing %C2 and %C3 but increasing %C4 suggested preferential removal of humic-like components, while %C1, %C2, BIX, and FI were generally less affected by photodegradation. Overall, this study revealed the difference in the photodegradation of CDOM/FDOM from different sources and the effects on the optical indices of DOM composition. The results have implications for both understanding the biogeochemistry of CDOM and the applications of optical indices in the field of DOM study.

## Figures and Tables

**Table 1:** Changes of optical indices by photodegradation.

Source	Time (h)	*a*_280_ (m^−1^)	*S*_275–295_ (nm^−1^)	*S*_R_	C1 (RU)	C2 (RU)	C3 (RU)	C4 (RU)	%C1	%C2	%C3	%C4	HIX	BIX	FI
Influent	0	8.0	0.0151	0.83	0.00	0.39	0.55	0.39	0.0	29.5	41.1	29.4	3.0	0.92	1.86
	60	5.5	0.0291	1.45	0.08	0.12	0.09	0.21	15.7	24.1	18.3	41.9	1.2	1.41	1.50
	%Change	−31	92	75	/ ^1^	−69	−83	−46	/ ^1^	−18	−56	43	−61	53	−19
Effluent	0	7.2	0.0119	0.68	0.00	0.34	0.51	0.30	0.0	29.5	44.4	26.0	4.0	1.01	1.86
	60	6.1	0.0193	2.30	0.06	0.11	0.10	0.15	13.5	27.3	23.8	35.3	1.7	0.90	1.58
	%Change	−16	62	237	/ ^1^	−66	−80	−50	/ ^1^	−7	−46	36	−58	−11	−15
Plant	0	14.1	0.0398	1.61	2.97	0.05	0.04	0.12	93.2	1.7	1.3	3.8	0.04	1.16	1.56
	60	12.9	0.0367	2.10	2.33	0.06	0.04	0.08	92.8	2.2	1.7	3.3	0.04	1.00	1.55
	%Change	−9	−8	31	−22	/ ^1^	/ ^1^	−32	0	/ ^1^	/ ^1^	−14	/ ^1^	−13	−1
Leaf litter	0	25.6	0.0123	0.66	0.00	0.67	0.42	0.13	0.0	55.2	34.4	10.4	12.2	0.53	1.32
	60	18.3	0.0200	1.23	0.06	0.31	0.20	0.15	8.2	43.3	28.2	20.3	3.5	0.53	1.15
	%Change	−28	63	86	/ ^1^	−53	−51	17	/ ^1^	−22	−18	96	−71	0	−13
River	0	6.4	0.0167	1.02	0.03	0.25	0.23	0.14	4.4	38.3	35.5	21.8	3.8	0.83	1.55
	60	4.8	0.0233	1.62	0.04	0.10	0.06	0.07	15.4	36.4	23.9	24.3	2.2	0.75	1.48
	%Change	−24	40	59	/ ^1^	−60	−72	−53	/ ^1^	−5	−33	12	−40	−10	−4

^1^ Not determined due to the lowest levels in the initial DOM.

## Data Availability

This published article includes all the data generated or analyzed during the study. The raw data of this research can be obtained by contacting the authors.
